# Identification of a novel NAMPT inhibitor by CRISPR/Cas9 chemogenomic profiling in mammalian cells

**DOI:** 10.1038/srep42728

**Published:** 2017-02-16

**Authors:** David Estoppey, Jeffrey W. Hewett, Chantale T. Guy, Edmund Harrington, Jason R. Thomas, Markus Schirle, Rachel Cuttat, Annick Waldt, Bertran Gerrits, Zinger Yang, Sven Schuierer, Xuewen Pan, Kevin Xie, Walter Carbone, Judith Knehr, Alicia Lindeman, Carsten Russ, Elizabeth Frias, Gregory R. Hoffman, Malini Varadarajan, Nadire Ramadan, John S. Reece-Hoyes, Qiong Wang, Xin Chen, Gregory McAllister, Guglielmo Roma, Tewis Bouwmeester, Dominic Hoepfner

**Affiliations:** 1 Novartis Institutes for BioMedical Research, Novartis Pharma AG, Forum 1 Novartis Campus, CH-4056 Basel, Switzerland; 2 Novartis Institutes for BioMedical Research, 250 Massachusetts Avenue, Cambridge, MA 02139, USA

## Abstract

Chemogenomic profiling is a powerful and unbiased approach to elucidate pharmacological targets and the mechanism of bioactive compounds. Until recently, genome-wide, high-resolution experiments of this nature have been limited to fungal systems due to lack of mammalian genome-wide deletion collections. With the example of a novel nicotinamide phosphoribosyltransferase (NAMPT) inhibitor, we demonstrate that the CRISPR/Cas9 system enables the generation of transient homo- and heterozygous deletion libraries and allows for the identification of efficacy targets and pathways mediating hypersensitivity and resistance relevant to the compound mechanism of action.

The recent increase in the number of new drug approvals can in part be explained by a shift in the pharmaceutical industry towards phenotypic assays as the basis for new programs. Patient-derived iPS cells, 3-D organoid cultures, tissue printing (just to name a few) allow for increasingly complex phenotypic assays with much higher physiological and disease relevance compared to experiments on isolated protein targets *in vitro*. However, one of the major challenges associated with compounds identified in such phenotypic screens still remains the elucidation of their binding target(s) and mechanism of action.

Chemogenomic profiling by haploinsufficiency (HIP) and homozygous profiling (HOP) has demonstrated to be a powerful and unbiased approach to gain insight into the biology modulated by bioactive probes^1^. HIP (based on heterozygous deletions and consequently ~50% decrease of protein levels) identifies pathways directly affected by the compound. HOP (both gene copies deleted) reveals synthetic lethality and identifies compensating pathways. Large-scale datasets as well as numerous individual applications of this approach have been published showing the impact and elegance of the approach^2^,^3^,^4^. However, due to the dependency on genome-wide, bar-coded deletion collections this approach has been mainly limited to *S. cerevisiae*. This report shows that chemogenomic profiling, including haploinsufficiency profiling of essential targets, may be deployed in a mammalian cellular context by taking advantage of the CRISPR/Cas9 system. Described here is a protocol that allows for the identification of the primary, efficacy target of bioactive compounds.

To generate targeted loss-of-function alleles with genome-wide coverage we deployed a lentiviral, guide RNA (sgRNA) library with a redundancy of 5 sgRNAs per gene following published design principles^5^. For this proof of concept study, a stable, Cas9-expressing human colorectal carcinoma cell line (HCT116) was generated. Chemogenomics depends on gene-copy number effects, we therefore chose the HCT116 line as it is described to be near diploid with a modality number of 45 (62%)^6^ and shows robust growth characteristics. Cas9-mediated editing of Cas9-expressing clones was then confirmed by showing disruption of the PIG-A locus using the FLAER assay to monitor inactivation^7^. The line was then transduced with the sgRNA pool at a multiplicity of infection around 0.5 and a coverage of 1000 cells/sgRNA.

In contrast to the specifically designed deletions in yeast, the CRISPR/Cas9 system does not allow for the controlled generation of heterozygous loss of function alleles. Nevertheless, heterozygous alleles are a prerequisite for a HIP assay that in yeast displays direct effects and thus is the relevant assay for target identification. We hypothesized that at least during the initial phase of editing, a given percentage of cells would be either heterozygous for a given gene or if homozygous, with some residual protein still present. Therefore, by conducting target identification during the phase of incomplete editing such an experiment would mimic the compound-induced hypersensitivity observed in the fungal HIP assay.

In this proof-of-concept study, we aimed at identification of the mechanism of action of a hit identified in a medium throughput phenotypic cancer screen. LB-60-OF61, a pyrrolopyrimidine compound from the Novartis archive without previous annotation was identified to potently inhibit proliferation of several cancer cell lines displaying high levels of expression and dependency on the MYC oncogene with certain selectivity against other cells. (Fig. 1a, Supplemental Data 1). Extended testing showed that LB-60-OF61 was also potent against HCT116 cells with an IC_50_ around 30 nM as measured in a 384 well format. For chemogenomic profiling compounds need to be carefully dosed at sub lethal concentrations thus we repeated the potency determination in 6 well plates and identified the IC_30_ to be 10 nM and the IC_50_ to be approximately 15 nM under these conditions. These concentrations were used in the genome-wide experiment that was conducted according to the scheme depicted in Fig. 1b. Special emphasis was taken to maintain complexity of the sgRNA pools above the 1000 sgRNA/cell threshold when splitting the cells. We took samples at day 14, 18, and 21 and processed these for analysis as described below. Plotting the RSA p-value (a gene-level measure for conserved depletion of its respective guides) against Q1 (a gene-level effect size corresponding to the RSA p-value)^8^ identified only a few genes that separated from the pool (Fig. 1c). In the 10 nM sample, the NAMPT gene, coding for the nicotinamide phosphoribosyl transferase, was the top scoring, underrepresented hit at all time-points with the best separation from the rest of the pool at day 14 (Fig. 1c). In the higher dose experiment, NAMPT collapsed back into the pool at the later time-points. NMNAT1, coding for nicotinamide nucleotide adenylyltransferase, the enzyme downstream of NAMPT in the NAD biosynthesis pathway was the second best hit and behaved similarly over time as measured by the quantile analysis.

We also computed RSA (-log p-value) for enrichment, representing sgRNA editing results in resistance to LB-60-OF61 treatment. It was apparent that duration and/or higher doses yielded more hits. This fits with the idea of homozygous profiling (HOP) where complete inactivation of genes reveals synthetic genetic interactions. Obvious hits at multiple time-points and doses include PARG (poly ADP-ribose glycohydrolase), Sirtuin 1 (SIRT1), phosphofructokinase (PFKP), aconitase (ACO2), and the E1A binding protein EP300. Inhibition of NAMPT has been previously shown to lead to NAD and energy depletion^9^. With the exception of EP300, the resistant hits all play a key role in NAD consumption, recycling or energy homeostasis. Thus, the identified resistance profile supported LB-60-OF61 being a NAMPT inhibitor.

When considering the underlying mechanisms potentially leading to the observed profiles, it became apparent that behavior of the top hit NAMPT was in agreement with the concept of haploinsufficiency profiling: i) as LB-60-OF61 was cytotoxic the target was expected to be essential; a characteristic reported for complete NAMPT inactivation in HCT116 cells^10^, ii) hypersensitivity of NAMPT-edited cells to LB-60-OF61 at early time-points suggested the existence of heterozygous cells due to incomplete editing iii) extended editing was expected to reduce heterozygous events and, as expected impaired, resolution at later time points, iv) detection of compound-induced haploinsufficiency required very defined IC concentration that favor cells with two intact copies^3^, potentially explaining better resolution of NAMPT at 10 nM.

To experimentally investigate if HIP was the underlying mechanism leading to separation of NAMPT from the other genes in the pool we transduced HCT116-Cas9 cells with the best scoring single sgRNA against NAMPT and harvested samples at the time points used to calculate the profiles shown in Fig. 1. To confirm time-dependent editing and existence of heterozygous cells we directly analyzed the mutational status of the NAMPT target locus by capturing single cells using the Fluidigm C1 instrument followed by single-cell sequencing. Mutations identified, were mostly found around the predicted Cas9 cut site, and a maximum of four haplotypes were identified per cell (See Supplemental Table S2). As expected for cells with constitutive active CRISPR/Cas9 components, we observed an increasing percentage of mutated NAMPT genes over time when analyzing cells at day 14, 18 and 21 (Fig. 2a). A low, constant level of cells with wildtype alleles was in line with the percentage of cells likely not transduced with the sgRNA construct as apparent by the flow-cytometry analysis on day 4. Next, we analyzed the type of mutations and the functional consequences on the NAMPT protein. The assayed sgRNA directs editing of first exome of the NAMPT gene. The NAMPT N-terminus was not annotated to contain distinct structural motifs, was not in the vicinity of the active site based on published crystal structures^11^ and no resistance conferring residues have been identified in this region. We thus annotated all alleles that contained silent mutations, indels that did not affect the start codon, the first exon/intron junction, nor led to frame-shifts as potentially functional. The total percentage of cells harboring alleles annotated as functional increased over time suggesting a selective proliferative advantage. This is in line with the reported essential role of NAMPT (Fig. 2b). At day 14, the percentage of heterozygous cells with both, functional and non-functional alleles was highest. As this was also the time-point with the best resolution for NAMPT in the profile shown in Fig. 1c, this provided support for haploinsufficiency as the underlying mechanism. We used Western blot analysis as an orthogonal method to measure NAMPT protein levels over time (Fig. 2c). In line with the analysis shown in Fig. 2b we found them lowest at day 14. Day 18 and 21 showed increasing levels but not reaching wild-type intensity as predicted by Fig. 2b.

Both, hypersensitive and resistant candidate genes of our chemogenomic profiling experiments supported NAMPT as the primary target of LB-60-OF61. We decided to validate this finding by three orthogonal methods: First, to enable competition-based chemoproteomics LB-60-OF61 was modified to ZA-87-IW08 to allow immobilization on NHS activated sepharose beads. Importantly, the N-acylated linker surrogate ND-37-YO30 retained the same cellular activity of LB-60-OF61, indicating that a ZA-87-IW08-based affinity matrix should be suitable for functional target enrichment. Quantitative mass spectrometry-based analysis, using iTRAQ isobaric mass tags, identified and robustly quantified 2327 proteins common to both experiments, with NAMPT showing strong enrichment as well as the strongest dose-responsive competition (Fig. 2d, Supplemental Fig. S1, Supplemental Table S3).

Second, inhibition of NAMPT has been reported to lead to NAD and consequently energy depletion^9^. Restoring NAD levels by addition of nicotinamide mononucleotide or nicotinic acid rescued this effect^9^. Supplementing the tissue culture medium with 10 μM nicotinic acid shifted the IC_50_ concentration of LB-60-OF61 by more than 150-fold but had no effect on the potency of the non-NAMPT inhibitor doxorubicin (Fig. 2e).

Third, distinct point mutations in NAMPT has been demonstrated to result in significant resistance against chemical inhibitors^12^. Among those mutations was H191R that interfered with inhibitor-binding by a steric clash and conferred >100 fold IC_50_ shifts against several chemical inhibitors^13^. We cloned the cDNAs of NAMPT_wildtype_ and the NAMPT_H191R_ mutant under control of the doxycycline-inducible promoter and transduced them into cells. Upon induction by doxycycline we observed a 240 fold IC_50_ shift with the mutant variant whereas induction of the wild-type had no effect (Fig. 2f). This suggested that resistance was dominant and supported a binding mode similar to previously reported NAMPT inhibitors. Following up this observation by *in silico* docking of LB-60-OF61 into the NAM binding cavity next to H191 outlined the potential of LB-60-OF61 to engage the catalytic site of NAMPT in a similar mode as published for other inhibitors (Supplemental Fig. S2).

In summary, independent validation by three different approaches supports NAMPT as the primary binding target of LB-60-OF61 as predicted by the CRISPR mediated chemogenomic profiling. LB-60-OF61 was identified as a cytotoxic compound with a selectivity towards MYC overexpressing cell lines. Interdependency between MYC levels and NAMPT have been outlined before and LB-60- LB-60-OF61 provides an additional tool to further dissect this interplay^14^,^15^,^16^.

In contrast to the other validation methods, chemogenomic profiling required neither chemical modification of the probe nor a pre-existing target hypothesis. Furthermore, it not only pinpointed the primary target but also provided information on synthetic genetic interactions with compensatory mechanisms. Pioneering RNAi-based methods already had supported the identification of a NAMPT inhibitor but the assay was not genome-wide and no resistance conferring hits could be identified in the same experiment^17^.

Recent work suggested that CRISPR was not a suitable tool for chemogenomic profiling of compounds that modulate essential targets^18^. Single cell sequencing was used to provide evidence that under the conditions used in this study it is possible to exploit the transient pool of heterozygous cells to allow haploinsufficiency profiling to identify essential gene targets. Hypersensitivity at early time-points thus points towards targets and pathways directly affected by compounds whereas resistance at late time-points outlines potential bypass and resistance mechanisms. Additional experiments using this method also have resulted in successful target identification of other compounds (Estoppey *et al*., manuscript in preparation) supporting broad applicability of this approach. As depicted in Fig. 2g the assay appears to be robust and tolerates further miniaturization allowing for easier handling and higher throughput. It thus will be interesting to see how the fast moving CRISPR community embraces this approach and expands its potential for the target identification of novel chemical probes.

## Additional Information

**How to cite this article:** Estoppey, D. *et al*. Identification of a novel NAMPT inhibitor by CRISPR/Cas9 chemogenomic profiling in mammalian cells. *Sci. Rep.* **7**, 42728; doi: 10.1038/srep42728 (2017).

**Publisher's note:** Springer Nature remains neutral with regard to jurisdictional claims in published maps and institutional affiliations.

## Supplementary Material

Supplementary Materials

Supplementary Table S1

Supplementary Table S2

Supplementary Table S3

## Figures and Tables

**Figure 1:**
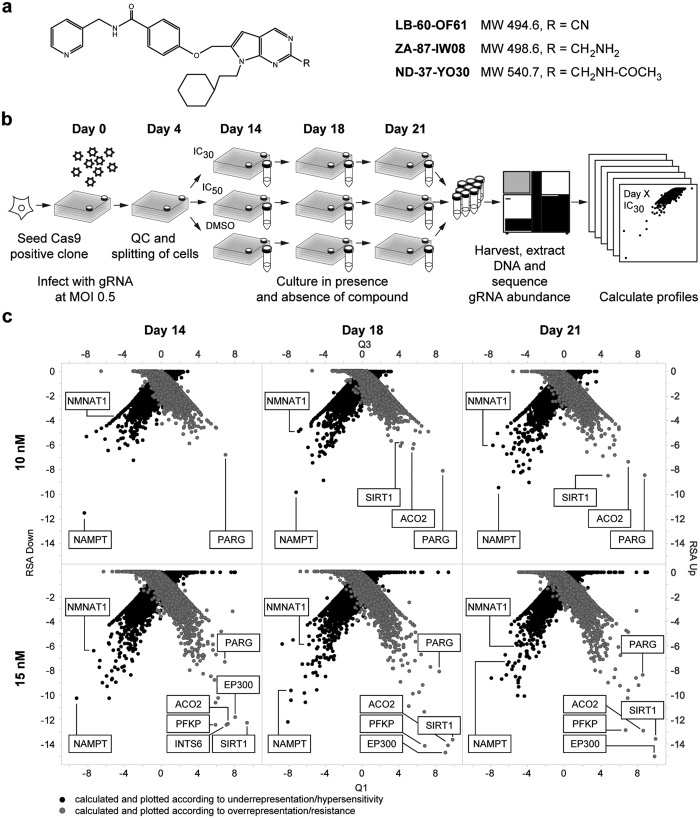
Chemogenomic profiling in mammalian cells by CRISPR/Cas9 mutagenesis. (**a**) Structure of LB-60-OF61, ZA-87-IW08 and ND-37-YO30 used in this study. (**b**) Schematic representation of the workflow. A HCT116-Cas9 clone was expanded to 200 million cells and transduced using a sgRNA library with genome-wide coverage (5 sgRNAs/gene). Cells were selected for successful transduction and at day 5 split into three branches: compound treatment at IC_30_, IC_50_ and DMSO control. Samples were collected at day 14, 18 and 21 and analyzed for relative sgRNA abundance by NGS. Profiles were then calculated as described in the methods section. (**c**) Results from the chemogenomic profiling experiment of LB-60-OF61 with special focus on optimal experimental parameters including timepoint and dosage. At the lower compound dose, we consistently identified the nicotinamide phosphoribosyl transferase NAMPT as the strongest hit following by NMNAT1, the next enzyme in the NAD biosynthesis pathway. At the higher dose these hits collapsed back into the pool over time. Resistant hits included major NAD consumers or key enzymes in energy metabolism (SIRT1, PFKP, ACO2, PARG, discussed in the text). Higher compound dose or longer testing appeared to favor identification of resistant hits whereas lower dose and shorter experiments yielded cleaner profiles for hypersensitive hits.

**Figure 2:**
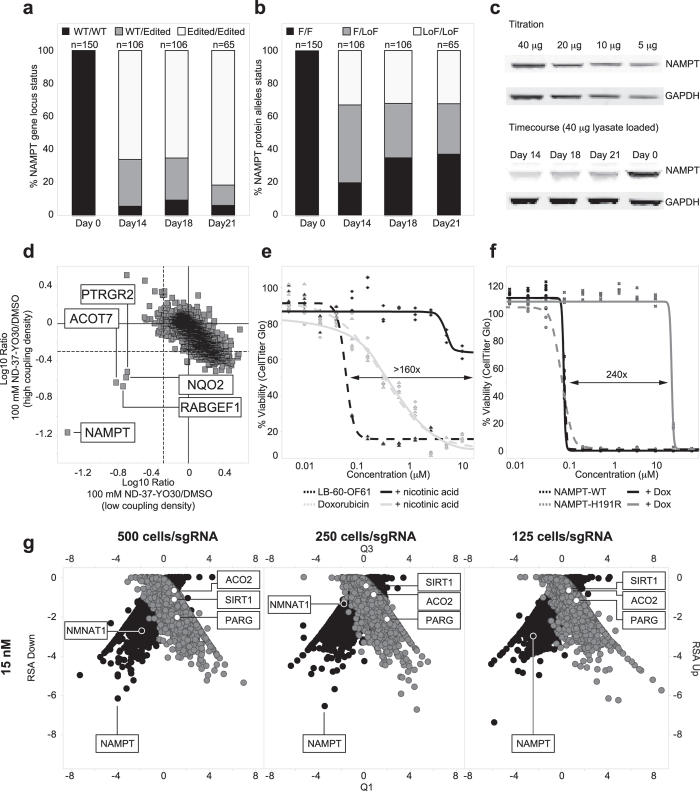
Time-dependent CRISPR/Cas9 editing of the NAMPT locus as analyzed by single-cell sequencing and validation of NAMPT as primary target of LB-60-OF61. (**a**) Time-dependent editing of the NAMPT locus by a single sgRNA analyzed by sequencing the genomic locus of single cells. WT = Wildtype, n = number of cells analyzed. (**b**) Functional effect on NAMPT protein as predicted by the identified genomic mutations in single cells. F = Functional, LoF = Loss of Function, n = number of cells analyzed. (**c**) Time-dependent effect on NAMPT protein levels in cell pools as observed by Western blot analysis. (**d**) Chemoproteomics using a LB-60-OF61 affinity matrix identified NAMPT as LB-60-OF61 binder: Scatter plot showing competition of binding to LB-60-OF61 affinity matrices with low (1 mM) and high 2 mM coupling density by 100 μM ND-37-YO30, respectively. Solid lines denote no competition (Log10 fold change 0), dashed lines denote 50% competition (Log10 fold change −0.3). (**e**) Addition of 10 μM nicotinic acid rescued growth inhibition by LB-60-OF61. (**f**) Induction of a NAMPT allele with a point mutation in the active site but not wildtype shifted the IC_50_ of LB-60-OF61. (**g**) Analysis of sampling size effects on data quality by repeating the chemogenomic profiling experiment with 500, 250 and 125 cells/sgRNA.
