# Supplementary material for: Identification of a novel NAMPT inhibitor by CRISPR/Cas9 chemogenomic profiling in mammalian cells
Source: Sci Rep. 2017 Feb 16;7:42728. doi: 10.1038/srep42728 (PMC5311948; doi:10.1038/srep42728)
Supplement: Supplementary Materials [file srep42728-s1.pdf]

Online supplemental materials for:

Identification of a novel NAMPT inhibitor by CRISPR/Cas9 chemogenomic profiling in mammalian cells

David Estoppey[a], Jeffrey W. Hewett[b], Chantale T. Guy[b], Edmund Harrington[b], Jason R. Thomas[b], Markus Schirle[b], Rachel Cuttat[a], Annick Waldt[a], Bertan Gerrits[a,c], Zinger Yang[b], Sven Schuierer[a], Xuewen Pan[b], Kevin Xie[b], Walter Carbone[a], Judith Knehr[a], Alicia Lindeman[b], Carsten Russ[b], Elizabeth Frias[b], Gregory R. Hoffman[b], Malini Varadarajan[b], Nadire Ramadan[b], John S. Reece-Hoyes[b], Qiong Wang[b], Xin Chen[b], Gregory McAllister[b], Guglielmo Roma[a], Tewis Bouwmeester[a], Dominic Hoepfner[a*]

[a]Novartis Institutes for BioMedical Research, Novartis Pharma AG, Forum 1 Novartis Campus, CH-4056 Basel, Switzerland

[b]Novartis Institutes for BioMedical Research, 250 Massachusetts Avenue, Cambridge, MA 02139, USA

[c]Current address: Department of Chemistry and Applied Biosciences, ETH Zurich, Vladimir-Prelogweg 3 8093, Zurich, Switzerland

[*]Corresponding author, dominic.hoepfner@novartis.com

Table of content

IDENTIFICATION OF LB-60-OF61 3

DERIVATIZATION OF LB-60-OF61 3

DETERMINATION OF LB-60-OF61 POTENCY 4

GENERATION AND CHARACTERIZATION OF THE HCT116-Cas9 CLONE 4

sgRNA LIBRARY DESIGN AND CONSTRUCTION 5

VIRAL PACKAGING 6

CHEMOGENOMIC PROFILING 6

DETERMINATION OF RELATIVE sgRNA ABUNDANCE BY NEXT GENERATION SEQUENCING 7

PROCESSING OF CHEMOGENOMIC PROFILING EXPERIMENT 8

SINGLE-CELL ANALYSIS OF THE NAMPT LOCUS 8

CHEMICAL PROTEOMICS 11

NAD RESCUE EXPERIMENT 16

IDENTIFICATION OF RESISTANCE CONFERRING MUTATIONS IN NAMPT 16

IN SILICO DOCKING APPROACHES 16

SUPPLEMENTAL REFERENCES 17

Identification of LB-60-OF61

The MYC (c-MYC) oncogene drives oncogenic transformation of cells and thus contributes to the genesis of many human cancers[1]. As increased expression and amplification of the MYC gene are a hallmark of oncogenic transformation we aimed at identifying compounds that inhibit proliferation of cells with high MYC activity. For this purpose, we selected four cell lines with high MYC activity and cellular MYC dependency and four cell lines with low MYC levels and low dependency on MYC. This cell panel was then used to screen for compounds that selectively caused cytotoxicity in the MYC high, MYC-dependent group only. Compounds were tested in 8-point dose-response fashion and viability was assessed by CellTiter-Glo assay according to the manufacturer's instructions (#G7573, Promega). Compound LB-60-OF61 was prioritized based on its greater selectivity toward the four MYC high/dependent cells lines (IC_{50} < 100 nM) versus the low/independent cell lines (IC_{50} >1 μM). IC_{50} stands for the concentration at which 50% growth inhibition was observed.

Cell lines for the screen were selected as follows: MYC expression was based on both quantitative PCR (qPCR) and protein quantification by western blot analysis (data not shown). Dependency was determined via knock down of MYC transcripts by transfecting four individual siRNA reagents targeting MYC, (Dharmacon, ON-TARGETplus MYC, # LU-003282-02-0002) and monitoring cell death using a luminescent cell viability assay (CellTiter-Glo, #G7570, Promega). This approach classified Hey-A8, A2780, KURAMOCHI, H1792 cells as high MYC/high dependency cells and Calu-6, Caov-3, COV318, H2070 cells as low MYC/low MYC dependency cells from the Cancer Cell Line Encyclopedia (CCLE) collection[2].

Derivatization of LB-60-OF61

Compound LB-60-OF61 is a deazapurine chemotype identified in the MYC cancer screen described above. Early structure-activity relationship (SAR) studies revealed the importance of the 3-pyridyl pendant portion of the compound. Subtle changes to this portion of the molecule were detrimental to compound activity so the 3-pyridyl substitution was retained for subsequent SAR optimization. Removal or reduction of the nitrile group preserved the MYC dependent cytotoxicity. This resulted in the identification of ZA-87-IW08, a potent molecule that retains MYC dependent cytotoxicity. ND-

37-YO30 and other derivatives with bulkier acyl groups (e.g. R = O-tButyl) demonstrated a general tolerance for large substituents at this position and therefore validated compound design for immobilization onto affinity beads at this position.

Determination of LB-60-OF61 potency

To correctly dose compounds for the chemogenomic profiling experiment, cytotoxic effects of LB-60-OF61, ZA-87-IW08 and ND-37-YO30 on HCT116 cells (#CCL-247, ATCC) were assessed in 384 well plates by seeding 1500 cells/well and adding serially diluted compound in a dose range from 0.37 nM to 20 μM. The compound solvent dimethyl sulfoxide (DMSO) was normalized to 0.2 %. 72 hours after compound addition cell viability was measured using the CellTiter-Glo assay according to the manufacturer's instructions (#G7573, Promega). Data was analyzed and IC_{50}s (compound concentration at which 50% inhibition was observed) calculated using the logistic regression curve fit analysis of the Tibco Spotfire package (version 6.5.3, TIBCO Software Inc.). As the LB-60-OF61 inhibition curve was very steep it was difficult to pinpoint the IC_{30} and IC_{50} concentrations. We thus repeated testing in 6 well plates, seeding 100'000 HCT116 cells/well testing a narrow dose range (0, 10, 20, 30, 40 and 100 nM) as directed by the 384 well experiment. 72 hours after compound addition viability was assessed using a Vi-cell XR Cell Viability Analyzer (Beckman Coulter) and curves plotted as described above. The IC_{30} and IC_{50} concentrations obtained from the 6 well experiments were then used for the chemogenomic profiling experiment.

Generation and characterization of the HCT116-Cas9 clone

The *Cas9* gene encoding the *S. pyogenes* **C**RISPR **as**sociated protein **9** RNA-guided DNA endonuclease Cas9[3] was cloned under control of the human cytomegalovirus (CMV) promoter into a lentiviral construct derived from pLenti6 (#V49610, Invitrogen) carrying a blasticidin resistance cassette. Upon packaging, the active virus was used to transduce the construct into HCT116 cells (#CCL-247, ATCC) grown in DMEM high glucose (#31966047, Life Technologies). Blasticidin-resistant clones were picked, analyzed for Cas9 expression and assessed for editing using a short

guide RNA (sgRNA) against PIG-A encoding an enzyme in the glycophosphatidylinositol (GPI) anchor biosynthesis pathway with the following sequence: 5'-TGGCGTGGAAGAGAGCATCA-3' by a genetic adaptation of the FLAER (Fluorescently, Alexa488-labeled, inactive variant of aerolysin) assay[4]. For this we infected cells with the PIG-A sgRNA and a control at a multiplicity of infection (M.O.I.) of 1. Cells were kept on puromycin selection until day four and transduction efficiency was assessed by flow cytometry using the red fluorescent protein (RFP) reporter encoded on the lentiviral construct. If >90% RFP positive cells were measured selective pressure was removed and the cells continued to be cultured. Decrease of GPI anchored proteins from the cell surface as an indirect measure of PIG-A editing/inactivation was measured by staining both control and PIG-A infected cells with the FLAER reagent (#FL2S, Cedarlanelabs) as follows: 100'000 cells/well in a fluorescence-activated cell sorting (FACS) compatible microwell plate were washed twice with PBS, and after removal of the supernatant 100 µl/well FLAER master mix added (1:100 diluted FL2S stock in 3% BSA) the cells gently resuspended and incubated in the dark at 37°C/5% CO_2 for 20 minutes. Then cells were washed twice in phosphate buffered saline (PBS), resuspended and analyzed by flow cytometry for percentage of stained/unstained cells. The control cells were used to set correct gating parameters. Decreasing levels of stained cells over time indicate functional editing and allow to characterize editing kinetics in the Cas9 positive cell line.

sgRNA library design and construction

The genome-wide sgRNA library targeting 18,360 protein-coding genes was constructed using chip-based oligonucleotide synthesis to generate spacer-tracrRNA-encoding fragments that were PCR-amplified and cloned as a pool into the BpiI site of the pRSI16 lentiviral plasmid (Cellecta). The modified tracrRNA scaffold published by Chen et al.[5] was used. Olfactory receptors were omitted from the library. The sgRNA designs were based on published sequences[6] and five sgRNAs were selected per gene targeting the most proximal 5' exons. 277 genes did not have published sgRNA sequence information and new sgRNAs were designed for these targets that contained an NGG PAM motif, filtering for GC content greater than 40% and less than 80%, eliminating homopolymer stretches greater then 4, and removing any guides with off-target locations having fewer than 4 mismatches across the

genome. Sequencing of the plasmid pool showed robust normalization with >90% clones present at a representation of +/- 5-fold from the median counts in the pool.

Viral Packaging

sgRNA libraries were packaged into lentiviral particles using HEK293T cells as described previously[7]. Packaging was scaled up by growing cells in CellSTACK flasks (#3313, Corning). For each cell stack, 2.1 $e10^7$ cells were transfected 24 h after plating using 510.3 µl of TransIT reagent (#MIR2300, Mirus) diluted in 18.4 ml of OPTI-MEM that was combined with 75.6 µg of the sgRNA libraries and 94.5 µg of lentiviral packaging mix (#CPCP-K2A, Cellecta; containing psPAX2 and pMD2 plasmids that encode Gag/Pol and VSV-G, respectively). 72 h post transfection, lentivirus was harvested, aliquoted, and frozen at -80°C. Viral titer was measured using the LentiX qPCR kit (#631235, Clonetech) and was typically in the range of 5.0 $e10^6$ TU/ml.

Chemogenomic profiling

For the profiling experiment one positive HCT116-Cas9 clone was expanded to 2.0 $e10^8$ cells and transduced with the lenti-viral sgRNA library (described in detail above) with a coverage of 5 sgRNAs/gene and a multiplicity of infection of 0.5. As described above, the library was designed as two sublibraries each covering 52'000 sgRNAs. Complexity of each subpool was kept always above at least 1000 cells/sgRNA. Each subpool was cultured in 1 CellSTACK flask (#3313, Corning) seeded with 6.7 $e10^7$ cells on day -1. On day 0 each CellSTACK was infected with one of the subpools. 4 Days after sgRNA infection and continuous selection on puromycin (2 µg/ml), transduction efficiency was assessed by flow cytometry and the experiment only continued if >95% cells were RFP positive. Cultures of each subpools were then split into 6 CellSTACK flasks at a cell density of 3.5 $e10^7$ cells. One day later LB-60-OF61 was added at an IC_{30} or IC_{50} to both subpool cultures, the control cultures (in duplicates as well) were treated with DMSO only and were used as time-matched controls for the subsequent analysis. DMSO concentration was adjusted to be equal in all cultures. Cultures were propagated and diluted again to 35mio cells when cells reached confluency (usually on day 7,11,14, 18). On day 14, 18 and 21, 7.0 $e10^7$ cells per condition were harvested and the genomic DNA was

extracted using the QIAamp DNA blood maxi kit according to manufacturer's instructions (#51194, Qiagen).

The experiment with reduced sgRNA/cell coverage was conducted as described above with the exception that in the 250 and 125 sgRNA/cell experiments the two subpools were combined and cultured in one CellSTACK flask. Each coverage experiment included a corresponding DMSO control experiment. Cells were split at the same time like the higher coverage experiments but with adjusted splitting ratios.

Determination of relative sgRNA abundance by next generation sequencing

Genomic DNA was quantified using Picogreen (#P11496, Invitrogen) following the manufacturer's recommendations. Illumina sequencing libraries were generated using PCR amplification with primers specific to the genome integrated lentiviral vector backbone sequence. A total of 24 independent PCR reactions were performed per 55,000 sgRNA transduced sample. PCR reactions were performed in a volume of 100 µl, containing a final concentration of 0.5 µM of each PCR primer (Integrated DNA Technologies, 5644 5'-AATGATACGGCGACCACCGAGATCTACACTCGATTTCTTGGCTTTATATATCTTGTGGAAAGGA-3' and INDEX 5'-CAAGCAGAAGACGGCATACGAGATXXXXXXXXXXGTGACTGGAGTTCAGACGTGTGCTCTTCCGATC-3', where the Xs denote a 10 base PCR-sample specific barcode used for data demultiplexing following sequencing), 0.5 mM dNTPs (#4030, Clontech), 1x Titanium Taq DNA polymerase and buffer (#639242, Clontech). PCR cycling conditions were as follows: 1x 98°C for 5 min; 28x 95°C for 15 sec, 65°C for 15 sec, 72°C for 30 sec; 1x 72°C for 5 min. The resulting Illumina libraries were purified using 1.8x SPRI AMPure XL beads (#A63882, Beckman Coulter) following the manufacturer's recommendations and qPCR quantified using primers specific to the Illumina sequences using standard methods. Illumina sequencing libraries were then pooled and sequenced with a HiSeq 2500 instrument (Illumina) with 1x 30b reads, using a custom read 1 sequencing primer 5645 (5'- TCGATTTCTTGGCTTTATATATCTTGTGGAAAGGACGAAACACCG-3'), and a 1x 11b index read, using the standard Illumina indexing primer (5'-

GATCGGAAGAGCACACGTCTGAACTCCAGTCAC-3'), following the manufacturer's recommendations. A total of 50-60 $e10^6$ reads were generated per transduced sample, resulting in an average of approximately a 1000 reads per sgRNA.

Processing of chemogenomic profiling experiment

Raw sequencing reads were converted to FASTQ format using Illumina bcl2fastq2 (Illumina bcl2fastq2 (Version 2.17.1.14); retrieved from http://support.illumina.com/downloads/bcl2fastq-conversion-software-v217.html). The reads were trimmed to the guide sequence using fastx-toolkit (Gordon, A and Hannon, G. FASTX Toolkit (Version 0.0.13); retrieved from http://hannonlab.cshl.edu/fastx_toolkit/index.html) and the trimmed reads were aligned to the sgRNA sequences in the plasmid library using Bowtie[8], with no mismatches allowed.

The R software package DESeq2[9] was used to evaluate differential sgRNA representation between the compound treated and the untreated samples. A robust z-score for each sgRNA was calculated using the median and mean-absolute deviation across the log2 fold changes of the library combined results. To summarize the results at the gene level we applied a methodology derived from siRNA screening analysis named redundant siRNA activity (RSA) analysis[10]. It models the probability of a gene 'hit' based on the collective activities of multiple siRNAs/sgRNAs per gene. All sgRNAs in our pool were initially ranked according to their individual signals. Then, the rank distribution of all 5 sgRNAs targeting the same gene was examined and a *P*-value was assigned. Thus, *P*-value indicates the statistical significance of all 5 sgRNAs targeting a single gene being unusually distributed toward the top (RSA up) or bottom (RSA down) ranking slots. To visualize the gene significance and result strength, we plot the RSA up value against the Q3 z-score for each gene for the investigation of gene deletion that promotes resistance and the RSA down value against the Q1 z-score for each gene for investigating genes that upon deletion increase sensitivity to the compound treatment. The **Supplemental Table S1** contains the annotated data for the all conducted experiments.

Single-cell analysis of the NAMPT locus

Time-dependent editing of the NAMPT locus by the CRISPR/Cas9 system was analyzed using a single sgRNA with the following sequence: 5'-GGCCAGGAGGATGTTGAACT-TRACR-3'. Of the five sgRNAs targeting NAMPT in the genome-wide pool this sgRNA resulted in the biggest Log2 fold change in the genome-wide profiling experiments described above. This sgRNA was transduced into 1.5 $e10^6$ cells as lentiviral construct at an M.O.I of 0.5, exactly as described in the genome-wide profiling experiment. Cells were selected on puromycin (2 µg/ml) and 4 days after sgRNA infection transduction efficiency was assessed by flow cytometry resulting in >95% RFP positive cells. Cells were cultured and samples for analyzes harvested at day 14, 18 and 21. For single cell analysis, cells were washed twice with PBS, detached with TrypLE (#12604013, ThermoFisher Scientific) and resuspended in tissue culture media. Prior to loading onto the integrated fluidics circuit plates (IFCs, #100-5763, Fluidigm) for DNA Seq (10-17 µm) cells were washed with PBS and adjusted to 250 cells per µl. The IFC plate was primed and loaded with cells according to the protocol PN100-7135 H1 from Fluidigm. A live/dead staining was done directly on the IFC plate according to the manufacturer's instructions. Each capture site (96/IFC plate) was imaged and scored for loading of a single, live cell. All wells not containing a single, live cell were not considered for subsequent analyses. The cDNAs for all 96 positions was harvested and diluted in 10 µl of C1 DNA Dilution Reagent, then stored at -20C for further processing. Two IFC plates were performed for day 14 and 18, only one IFC plate for day 21.

The genomic NAMPT locus was then amplified for each sample using the QIAGEN Multiplex PCR kit (#206145, Qiagen) together with the following 8 base pair barcoded primers:

NAMPT_FWD2_bc_A_GATCTCTC GATCTCTCcccgctcctcctcatctg

NAMPT_FWD2_bc_B_ATATCGCT ATATCGCTcccgctcctcctcatctg

NAMPT_FWD2_bc_C_TAGAGATA TAGAGATAcccgctcctcctcatctg

NAMPT_FWD2_bc_D_AGCGATAG AGCGATAGcccgctcctcctcatctg

NAMPT_FWD2_bc_E_TCGAGCGA TCGAGCGAcccgctcctcctcatctg

NAMPT_FWD2_bc_F_CTAGATAT CTAGATATcccgctcctcctcatctg

NAMPT_FWD2_bc_G_GATCGCGA GATCGCGAcccgctcctcctcatctg

NAMPT_FWD2_bc_H_CGAGCGAG CGAGCGAGcccgctcctcctcatctg

NAMPT_REV2_bc_1_GCGCTCGA GCGCTCGAccgaccgagcagtgactta

NAMPT_REV2_bc_2_AGATAGAG AGATAGAGccgaccgagcagtgactta

NAMPT_REV2_bc_3_TATCTCTC TATCTCTCccgaccgagcagtgactta

NAMPT_REV2_bc_4_CTATCTAG CTATCTAGccgaccgagcagtgactta

NAMPT_REV2_bc_5_GAGCGATC GAGCGATCccgaccgagcagtgactta

NAMPT_REV2_bc_6_ATAGAGCG ATAGAGCGccgaccgagcagtgactta

NAMPT_REV2_bc_7_GCTATCGC GCTATCGCccgaccgagcagtgactta

NAMPT_REV2_bc_8_CGCTCTCG CGCTCTCGccgaccgagcagtgactta

NAMPT_REV2_bc_9_TCGCTCTA TCGCTCTAccgaccgagcagtgactta

NAMPT_REV2_bc_10_CGCGATCT CGCGATCTccgaccgagcagtgactta

NAMPT_REV2_bc_11_TCTCGATC TCTCGATCccgaccgagcagtgactta

NAMPT_REV2_bc_12_AGAGATCG AGAGATCGccgaccgagcagtgactta

The combination of these primers provides 96 uniquely barcoded PCR products, one for each Fluidigm 96 IFC single cell DNA sample. The quality of the 96 PCR products was assessed on the TapeStation 4200 platform (Agilent) using a D1000 kit (#5067-5582, Agilent). The 96 amplicons were then pooled together at equal volumes and sequencing libraries were prepared with the Ovation Low complexity Sequencing System starting from each individual pool (#9092-256, NuGEN). Libraries were sequenced in paired-end mode, at a read length of 2 × 200 bp, using the MiSeq platform (Illumina).

The resulting FASTQ files were demultiplexed using the barcoded primers and the demultiplexed FASTQ files were aligned against the human reference genome (GRCh38) using BWA version 0.7.4[11] with the parameter setting: -t 1 -A 4 -B 16 -O 24 -E 1 -T 120 -L 20 to allow for alignments with long indels. The alignments were then processed by a custom script to extract all putative variants. Variants that were inconsistent between the two reads of a read pair were excluded. The mutations were then filtered based on the following criteria: The position of the variation had to be within ± 50bp of the sgRNA alignment, the coverage had to be at least 100, the number of reads containing the

variation at least 10, and the percentage of reads containing the variation at least 5% of the reads covering the position. The resulting variants were then aggregated into haplotypes based on the reads in which they occurred together. Reads without a variation were assigned to the wild-type (WT) haplotype. Based on the inspection of the data for the empty wells of the C1 IFC plate, we excluded haplotypes with coverage of less than 2048 for all IFC plates except for the first replicate of day 18 for which we reduced the coverage threshold to 512 due to the low sequencing depth of this experiment. Furthermore, we also excluded haplotypes which contained less than 15% of the reads covering the NAMPT locus. This resulted in one to four haplotypes per cell. Each haplotype was then manually classified by its functional consequence on the gene product as WT, functional, or non-functional. Finally, the functional classifications of the haplotypes of a cell were manually combined into a functional classification for the cell.

The raw sequencing reads are available in the NCBI Short Read Archive under the accession number SRP093844. The identified mutations and the functional consequences are summarized in the **Supplemental Table S2**.

Chemical proteomics

For synthesis of the ZA-87-IW08-derivatized resin, 2 ml packed resin volume of NHS-activated sepharose 4 fast flow (#17-0149-01, GE Healthcare) was washed 3 times with 10 ml anhydrous DMSO (#276855, Sigma-Aldrich). 4 or 8 ml of a 0.5 mM solution of ZA-87-IW08 (freshly prepared with anhydrous DMSO) was added to the NHS-activated sepharose resin along with 30 µl triethylamine (#90337, Sigma-Aldrich). The reaction mixtures were vortexed to mix and spun at 100 x g for 2 min. 50 µl aliquots of the supernatants were saved for LC/MS analysis. The reaction mixtures were incubated overnight at room temperature with end-over-end rotating agitation. The following day, the reaction mixtures were spun at 100 x g for 2 min. Again, 50 µl aliquots of the supernatants were saved for LC/MS analysis. Completion of coupling was inferred by loss of starting material following LC/MS analysis. 100 µl of 2-(2-aminoethoxy)ethanol (#A54059, Sigma-Aldrich) was added to the reaction mixtures, vortexed, and allowed to incubate overnight at room temp with end-over-end agitation. Finally, the resins were washed 3 times with 10 ml anhydrous DMSO.

Frozen cell pellets of A2780 cells were gently thawed on ice. Once thawed, the cell pellet was resuspended in Lysis Buffer (50 mM HEPES (pH 7.4), 150 mM NaCl, 1.5 mM MgCl2, 5% glycerol (v/v), 1 mM DTT, 0.8% NP40 + 1x HALT Protease Inhibitor Cocktail (#78430, Thermo Scientific)) with a volume that was 2 times the cell pellet volume. After a 30 min incubation on ice, the resuspended cells were subjected to dounce homogenization with 10 strokes of the tight fitting pestle and the resulting lysate was centrifuged at 800 x g for 10 min at 4°C. The supernatant (S0.8 fraction) was collected and saved. The pellet (P0.8) was resuspended in Low Salt Buffer (20 mM HEPES, 25% glycerol (v/v), 1.5 mM MgCl2, 0.2 mM EDTA, 1 mM DTT + 1x HALT Protease Inhibitor Cocktail) in a volume that was 0.5 times the pellet volume. High Salt Buffer (20 mM HEPES, 25% glycerol (v/v), 1.5 mM MgCl2, 2.4 M NaCl, 0.2 mM EDTA, 1 mM DTT + 1x HALT Protease Inhibitor Cocktail) was added dropwise to the resuspended nuclear pellet; the volume of High Salt Buffer added was equal to that of Low Salt Buffer. The resuspended P0.8 pellets were further processed using a Barocycler® NEP2320 (Pressure BioScience Inc.) (5 cycles: 35,000 psi for 20 sec followed by atmospheric pressure for 20 sec). Benzonase (#E8263, Sigma-Aldrich) was added to the resulting nuclear lysate at a final concentration of 90 U/ml. The nuclear lysate was allowed to incubate overnight at 4°C with gentle agitation, then clarified by centrifugation at 20,000 x g for 20 min at 4°C. This supernatant was combined with the S0.8 fraction to yield the final whole cell lysate used in affinity enrichment experiments.

For each affinity enrichment condition, 1 ml of A2780 lysates (5 mg) was preincubated with varying concentrations of ND-37-YO30 or DMSO control for 1 hr at 4 °C. During this pre-incubation, the ZA-87-IW08-derivatized sepharose beads (35 µl per sample) were washed 3 times with 3 ml Wash Buffer 2 (50 mM HEPES (pH 7.4), 150 mM NaCl, 1.5 mM MgCl2, 5% glycerol (v/v), 1 mM DTT, 0.4% NP40). Preincubated lysates were then incubated with ZA-87-IW08-derivatized resin for 4 hrs at 4 °C with end-over-end agitation. The beads were transferred to individual columns (MoBiTec), washed with 3 ml Wash Buffer 2, 1.5 ml Wash Buffer 2, followed by 1.5 ml Wash Buffer 1 (50 mM HEPES (pH 7.4), 150 mM NaCl, 1.5 mM MgCl2, 1 mM DTT). To elute bound proteins, 50 µl 2x LDS sample buffer (NuPAGE) + 10 mM DTT was added to each sample, which were incubated at 55

°C for 30 min. Eluted proteins were separated from resin by centrifugation (14,000 x g for 2 min at room temp). Proteins were alkylated with 200 mg/ml iodoacetamide for 30 min.

Proteins were subjected to in-solution trypsinization over night at 37ºC followed by isobaric labelling using iTRAQ 4plex reagents (#4466096, AB Sciex) using 114/115/116 for 100/10/1 µM ND-37-YO30 and 117 for the DMSO control. Samples were mixed and separated using high pH reverse phase chromatography (Dionex Ultimate 3000 HPLC, Waters Xbridge column (1 mm x 15 cm), mobile phase A: 100% H_2O; mobile phase B: 100% acetonitrile; mobile phase C (modifier, constant at 10%): 200mM ammonium formate, pH10; flow rate: 250ul/min, 60 min effective gradient). Fractions were pooled to 16 samples which were analyzed by nanocapillary liquid chromatography-tandem mass spectrometry on an Eksigent 1DC system coupled to a LTQ-Orbitrap Elite mass spectrometer (Thermo Scientific), using an in-house fabricated 75 µm ID spraying capillary packed with ReproSil-Pur 120 C18-AQ, 3 µm material (Dr. Maisch GmbH; 150 mm bed length) and a trapping column set-up (1cm Michrom Magic C18AQ, 5 µm). Peptides were eluted with a gradient of 3% Buffer B (70% acetonitrile in 0.1% formic acid) to 45% B in 80 min (0.5%B/min) delivered at a flow rate of 300 nl/min. Peptide mass and fragmentation data acquired by higher energy collisional dissociation (HCD) were searched against a combined forward-reverse IPI human database using Mascot (Matrix Science, IPI human v3.87 plus typical lab contaminants). Precursor and fragment ion tolerances were set to 10 ppm and 0.1 Da, respectively, allowing for 2 missed tryptic cleavages. Carbamidomethyl (C) was selected as fixed modification and iTRAQ4plex (K), iTRAQ4plex (N-term), Oxidation (M) as variable modifications. Peptide and protein validation was done using Transproteomic pipeline v3.3sqall (Institute for Systems Biology; http://tools.proteomecenter.org/software.php) using a false positive threshold of <1% for protein identifications and removing obvious contaminants. For each peptide sequence and modification state, reporter ion signal intensities from all spectral matches were summed for each reporter ion type and corrected according to the isotope correction factors given by the manufacturer. Only peptides unique to a given protein within the total dataset of identified proteins were used for relative protein quantification. Peptide fold changes were calculated (treatment over DMSO control) and subsequently renormalized within each replicate analysis using the median fold change of all quantified peptides to

compensate for differences in total protein yield for each affinity purification. Protein fold changes were calculated as median peptide fold change and p-values were calculated using a one-way T-test (arbitrarily set to 1 for non-significant single peptide quantitations) and adjusted using the Benjamini-Hochberg False Discovery Rate (FDR). Data were visualized for further analysis using TIBCO Spotfire v3.2.1. Figure 2d depicts the two experiments plotted as Log10 fold change over DMSO control for 2327 human proteins (distinct protein accession numbers) quantified based on 2 or more peptides with reporter ion intensities in all iTRAQ channels in both replicates. Solid lines denote no competition (Log10 fold change 0), dashed lines denote 50% competition (Log10 fold change -0.3). Full dose response quantitative proteomics data for all proteins quantified in the two experiments are given in **Supplemental Table S3**.

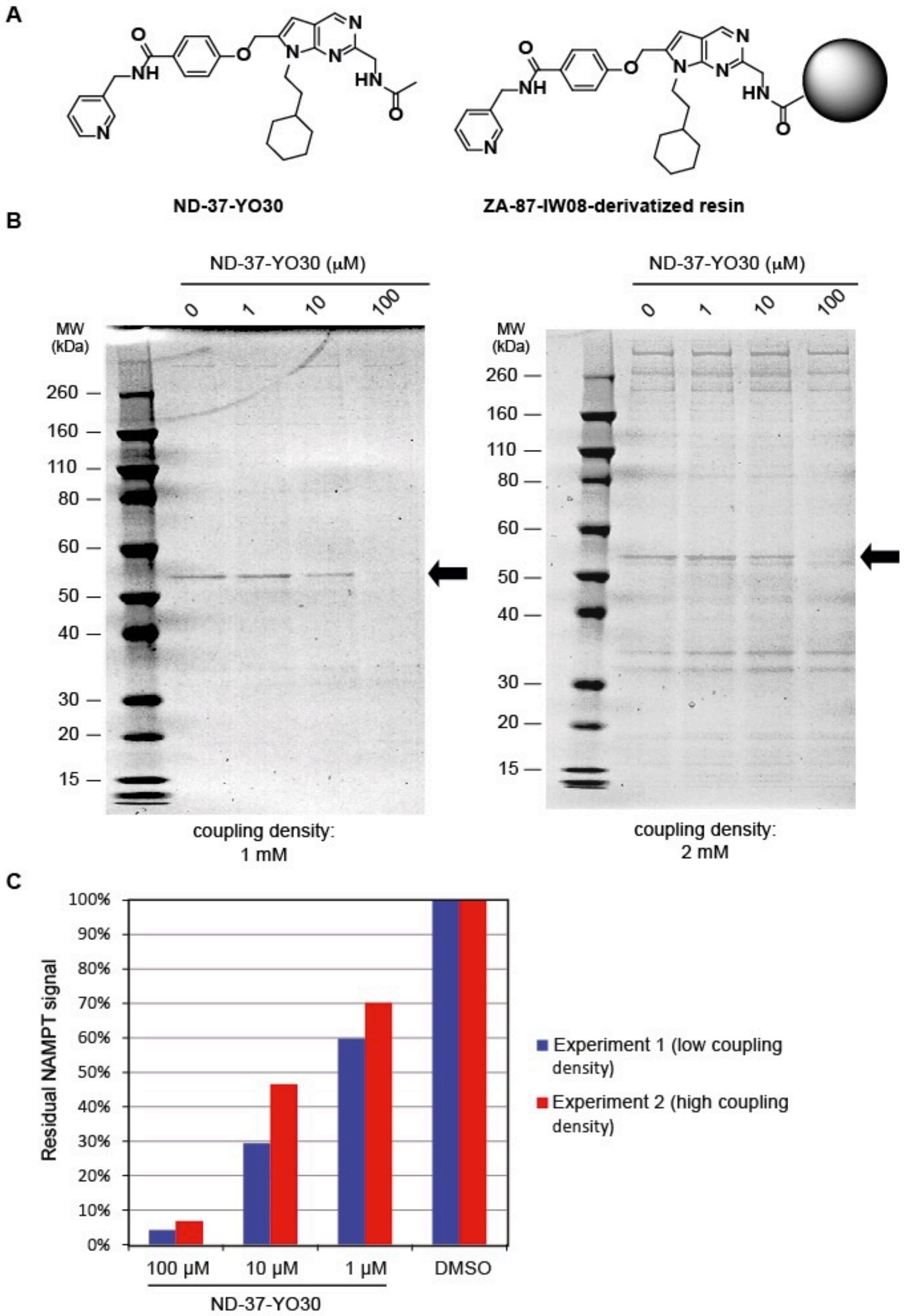

Supplemental Figure S1. Gel-analysis of lysates from chemoproteomics experiments. A) ZA-87-IW08 and ND-37-YO30 used in the chemoproteomics experiments; for ZA-87-IW08 the actual affinity resin after immobilization on NHS-activated sepharose is depicted. **B)** Gel separation of 10%

of each individual pulldown material identifies a strongly enriched protein in the 55kDa range (arrow) that shows visible competition with free ND-37-YO30. The molecular weight of the band corresponds to that of NAMPT (55521 Da). Taken together with the fact that NAMPT is among the most abundant proteins in the pulldown material based on semi-quantitative spectrum count (see Table S3), these data indicate that he ZA-87-IW08-based resin is able to strongly enrich functional NAMPT protein. **C)** Densiometric analysis of the bands from panel **B**. Decreasing intentsties of the NAMPT bands in presence of increasing concentrations of ND-37-YO30 indicates dose responsive competition by ND-37-YO30 across the two replicates.

NAD rescue experiment

Serially diluted compound (1:2 dilution factor) was dispensed into 384 well plates containing 1'500 cells/well prefilled with 40 µl growth medium containing supplemented with or without 10 µM nicotinic acid (#N4126, Sigma-Aldrich). The experiment was set up to obtain triplicate data points. Plates were incubated for 48 hours followed by cell viability determination using CellTiter-Glo (#G7570, Promega) according to manufacturer instructions.

Identification of resistance conferring mutations in NAMPT

The cDNA of wildtype or H191R mutant NAMPT was cloned into a pLENTI6-derived expression construct (#V49610, ThemrmoFischer Scientific) under control of the doxycycline-inducible promoter and packaged into infectious viral particles. Cells were transduced at a multiplicity of infection (M.O.I.) of 0.5 and selected on blasticidin. Blasticidin-resistant cells were seeded into 384 well plates at a density of 8000 cells/well prefilled with 90 µl/well growth medium (RPMI+10%FBS+Pen/Strep). Serially diluted compound (1:2.5 dilution factor) was dispensed and to a subset of plates 5ug/ml doxycycline added. The experiment was set up to obtain triplicate data points. Plates were incubated for 72 hours followed by cell viability determination using CellTiter-Glo (#G7570, Promega) according to manufacturer instructions.

***In silico* docking approaches**

LB-60-OF61 was analyzed for its potential to engage the catalytic pocket of the published NAMPT crystal structure (PDB code : 2GVJ[12]) by *in silico* docking approaches the using the Glide program (Schrödinger, LLC, New York, NY 10036, USA). The proposed docking solution and the key

interaction residues are presented in **Supplemental Figure S2** below. Histidine 191 that was identified to lead to significant resistance when mutated (**Figure 2f**) lies directly adjacent to the benzyl moiety of the LB-60-OF61 backbone and mutation to arginine would lead to a steric clash just like shown for the published NAMPT inhibitor GNE-618[13].

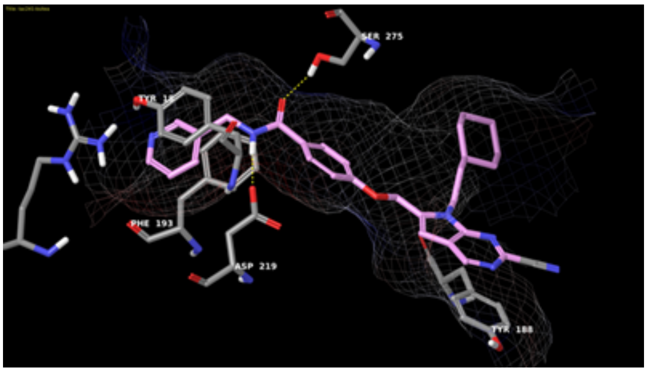

Supplemental Figure S2. Illustration of the docking model of compound1 bound in the NAMPT protein. The binding pocket is indicated by mesh surface. The ligand and its key interaction residues are represented in stick model and colored in pink and grey, respectively

Supplemental references

1 Dang, C. V. MYC on the path to cancer. *Cell* **149**, 22-35 (2012).

2 Barretina, J. *et al.* The Cancer Cell Line Encyclopedia enables predictive modelling of anticancer drug sensitivity. *Nature* **483**, 603-607 (2012).

3 Doudna, J. A. & Charpentier, E. Genome editing. The new frontier of genome engineering with CRISPR-Cas9. *Science* **346**, 1258096 (2014).

4 Brodsky, R. A. How I treat paroxysmal nocturnal hemoglobinuria. *Blood* **113**, 6522-6527 (2009).

5 Chen, B. *et al.* Dynamic imaging of genomic loci in living human cells by an optimized CRISPR/Cas system. *Cell* **155**, 1479-1491 (2013).

6 Wang, T., Wei, J. J., Sabatini, D. M. & Lander, E. S. Genetic screens in human cells using the CRISPR-Cas9 system. *Science* **343**, 80-84 (2014).

7 Hoffman, G. R. *et al.* Functional epigenetics approach identifies BRM/SMARCA2 as a critical synthetic lethal target in BRG1-deficient cancers. *Proc Natl Acad Sci U S A* **111**, 3128-3133 (2014).

8 Langmead, B., Trapnell, C., Pop, M. & Salzberg, S. L. Ultrafast and memory-efficient alignment of short DNA sequences to the human genome. *Genome Biol* **10**, R25, (2009).

9 Love, M. I., Huber, W. & Anders, S. Moderated estimation of fold change and dispersion for RNA-seq data with DESeq2. *Genome Biol* **15**, 550 (2014).

10 Konig, R. *et al.* A probability-based approach for the analysis of large-scale RNAi screens. *Nat Methods* **4**, 847-849 (2007).

11 Li, H. & Durbin, R. Fast and accurate short read alignment with Burrows–Wheeler transform. *Bioinformatics* **25**, 1754-1760 (2009).

12 Khan, J. A., Tao, X. & Tong, L. Molecular basis for the inhibition of human NMPRTase, a novel target for anticancer agents. *Nat Struct Mol Biol* **13**, 582-588 (2006).
13 Wang, W. *et al.* Structural basis for resistance to diverse classes of NAMPT inhibitors. *PloS one* **9**, e109366, doi:10.1371/journal.pone.0109366 (2014).
